# Fistules carotido-caverneuses post-traumatiques à propos d'un cas et revue de la littérature

**DOI:** 10.11604/pamj.2015.21.290.6210

**Published:** 2015-08-20

**Authors:** Jamal Oumellal, Olivier Bekaert, Sophie Gallas, Caroline Leguerinel, Stéphane Palfi, Fahd Derkaoui, Nizar El Fatemi, Rachid Gana, Moulay Rachid Elmaquili, Najia Elabbadi

**Affiliations:** 1 Service de Neurochirurgie et Neuroradiologie Interventionnelle, Hôpital Henri Mondor, Créteil, France; 2 Service de Neurochirurgie, Hôpital Avicenne, Rabat, Maroc

**Keywords:** Exophtalmie, fistule, embolisation, Exophtalmia, fistula, embolization

## Abstract

Les auteurs rapportent une observation clinique d'une fistule carotidocaverneuse survenue à la suite d'un traumatisme craniofaciale grave. Une exophtalmie unilatérale pulsatile et asymétrie des 2 sinus caverneux au scanner ont permis de suspecter le diagnostic. Une artériographie a permis de confirmer ce diagnostic, avec embolisation couronnée de succès chez ce malade, mais l’évolution est défavorable sur le plan neurologique avec apparition d'une HTIC réfractaire au traitement médical maximal. La fistule carotido-caverneuse est une complication rare mais grave pouvant engager le pronostic fonctionnel (cécité) et vital (hémorragie méningée et intracérébrale).L'artériographie et l'embolisation en un seul temps ont considérablement amélioré le pronostic.

## Introduction

Les fistules carotidocaverneuses post traumatiques sont des communications anormales entre le système carotidien et le sinus caverneux suite à un traumatisme craniofaciale grave. C'est une complication rare, mais non exceptionnelle des traumatismes craniofaciaux dont le diagnostic est suspecté en clinique. La situation profonde du sinus caverneux rend le traitement chirurgical difficile. Le pronostic s'est largement amélioré ces 20 dernières années grâce aux progrès de la neuroradiologie interventionnelle. Le but de ce travail est de présenter les premiers signes cliniques et para cliniques qui peuvent orienter ver le diagnostic de FCC, l'intérêt de la rapidité et l'efficacité du traitement endovasculaire et leurs impacts sur le pronostic vitale.

## Patient et observation

Un jeune homme de 29 ans, sans antécédent, il a reçu un tronc d'arbre sur le crane occasionnant un traumatisme crânien grave d'emblée et une perte de connaissance initiale, patient admis en réanimation chirurgicale avec score de Glascow 6, en anisocorie avec mydriase à droite,une exophtalmie modéré de l'oeil droite, présence d'une otorragie droite et une épistaxis qui était rapidement méchée, le reste de l'examen clinique, notamment thoraco-abdominal, et hémodynamique est normal.

**Figure 1:**
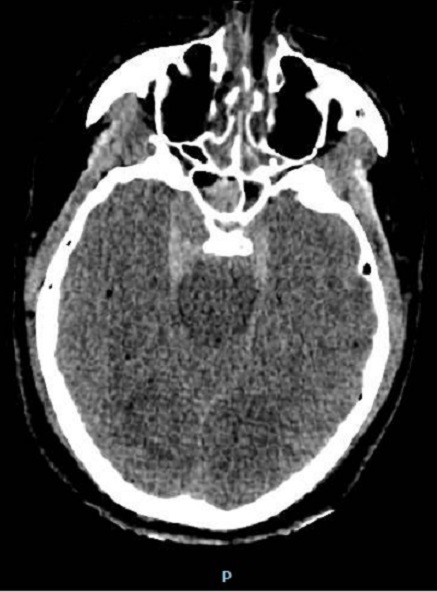
TDM cérébrale montre une hémorragie méningée, asymétrie des 2 sinus caverneux

**Figure 2:**
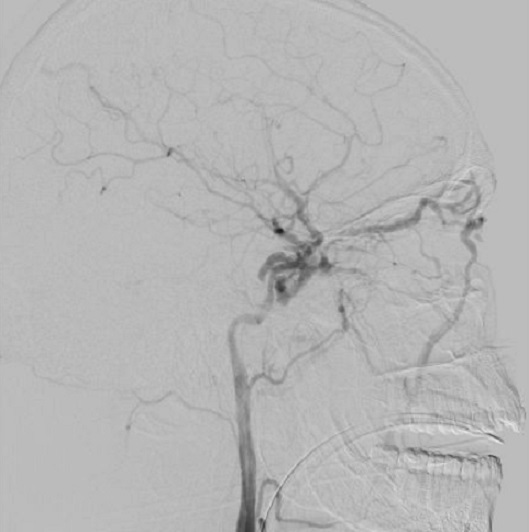
Artériographie carotidienne gauche montrant au temps artériel une fistule carotido-caverneuse avec opacification précoce des sinus caverneux

**Figure 3:**
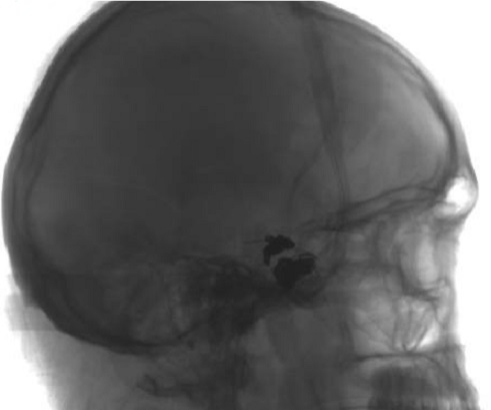
Artériographie carotidienne après embolisation montrant les ballonnets au niveau de la carotide interne

Le scanner cérébrale trouve hémorragie méningée avec asymétrie des 2 sinus caverneux, fracture du trou carotidien droit, fracture bilatérale du rocher, pneumocéphalie de la région sellaire, et comblement du sinus sphénoïdale (Figure 1). Ces signes ont fait douter une FCC, une artériographie (Figure 2) et une embolisation a été réalisé avec exclusion de FCC par des ballonnets largables, avec une bonne perfusion de la carotide interne droite le contrôle angiographique (Figure 3) a objectivé une occlusion complète de la fistule avec une bonne perfusion de la carotide interne droite perméable et une bonne circulation au niveau du polygone de Willis.

L’évolution défavorable sur le plan neurologique avec apparition d'une HTIC réfractaire au traitement médicale, faisant réaliser un scanner cérébral qui montre un oedème cérébral diffus, apparition d'ischémie dans les deux hémisphères, malgré la pose de DVE, le patient passe en mydriase bilatérale aréactive, un angioscanner confirme l'arrêt circulatoire intracérébrale et confirme le diagnostic de mort cérébrale.

## Discussion

La fistule carotidocaverneuse est un shunt artério-veineux anormale entre le système carotidien et le sinus carverneux. Elle est le plus souvent unilatérale, cependant quelques cas de formes bilatérales ont été décrites. Dans les suites d'un traumatisme crânien, on observe surtout les fistules artérioveineuses directes à débit élevé, type A de la classification de Barrow [1].

**Classification angiographique des fistules carotidocaverneuse selon Barrow et al., 1985**

**Type A:** shunt direct entre l'ACI et le sinus caverneux; **Type B:** shunt dural entre les branches méningées de l'ACI et le sinus caverneux; **Type C:** shunt dural entre les branches méningées de l'ACE et le sinus caverneux; **Type D:** shunt dural entre les branches méningées provenant à la fois de l'ACE et de l'ACI et le sinus caverneux. ACI: artère carotide interne; ACE: artère carotide externe.

La fistule entraine une communication anormale du flux artériel vers le sinus caverneux et ses affluents.ces structures sont inappropriées pour contenir un sang circulant à haut débit et à haut pression. On observe une dilatation du réseau veineux d'amont avec artérialisation [2]. Les signes cliniques oculaire sont prépondérants du fait de la position d'amont des veines ophtalmiques par rapport au sinus caverneux. Une exophtalmie pulsatile est retrouvée dans 90% des cas, associé à une baisse de l'acuité visuelle dans 80% des cas [3]. (Mais parfois l'exophtalmie est minime et non pulsatile, difficilement trouvable à l'examen clinique).

L'auscultation de la région périorbitaire et temporale retrouve un souffle intracrânien systolo-diastolique disparaissant à la compression manuelle de l'artère carotide homolatéral au niveau du cou [4]. (Cette symptomatologie peut être absente ou bien méconnue dans les grands fracas cranio-faciaux avec oedème important du visage et la FCC sera révélée plusieurs mois, voire plusieurs années après le traumatisme par des céphalées, des manifestations ophtalmologiques ou bien par une hémorragie cérébrale ou sous arachnoïdienne).

L'exploration neuroradiologique constitue un temps essentiel dans le diagnostic et le traitement des FCC post-traumatiques. L’écho-doppler couleur permet d'affirmer la fistule en montrant au niveau des veines ophtalmiques un signal doppler inversé dirigé vers la face à renforcement systolique. Cet permet en outre un suivi après embolisation ou abstention thérapeutique. Le doppler transcranien visualise directement la fistule avec une sensibilité de 95%(mais il est opérateur dépendants).

La tomodensitométrie cérébrale recherche le plus souvent les signes indirects, qui sont ipsilatéraux à la fistule ou parfois bilatéraux: élargissement du sinus caverneux et de la veine ophtalmique supérieure, infiltration des muscles oculomoteurs et des tissus orbitaires.

L'artériographie cérébrale est l'examen de certitude de FCC, et surtout mise en oeuvre des thérapeutiques dans le même temps interventionnel. Hmamouchi et al.préconisent même sa réalisation d'emblée devant une exophtalmie pulsatile associée à un souffle orbitaire systolo-diastolique [5].

L’évolution est marquée par troubles ophtalmologique et neurologique, sur le plan neurologique, il a été observé des complications secondaires aux FCC à partir d'une série de 155 patients [6]. Les plus fréquentes sont la varice du sinus caverneux, le drainage veineux cortical non physiologique, potentiellement responsable d'une hypertension intracrânienne et d'hémorragie intracrânienne, le pseudo anévrysme post-traumatique et l’épistaxis massive. Enfin, une résolution spontanée de ces FCC est démontrée dans 5 à 10% des cas dans une série de 132 patients, dans notre étude après le traitement de FCC [6] (le patient a présenté une hypertension intracrânien sévère réfractaire au traitement médicale, probablement en rapport avec son état neurologique initial défavorable (GCS à 6) aggravé par l'embolisation précoce; **donc faut-t-il traiter précocement les patients présentant des FCC post traumatiques ou préféré un traitement tardif après stabilisation de l’état clinique du malade?**.

## Conclusion

La FCC post traumatique est une complication rare mais grave pouvant engager le pronostic fonctionnel ou vital, leur diagnostic clinique est évoqué sur des signes ophtalmiques et orbitaires qui sont à rechercher activement chez tout traumatisé crânien. La surveillance quotidienne par le doppler transcranien peut permettre leur dépistage précoce et indiquer des explorations complémentaires. La visualisation des signes directs ou indirects sur le scanner cérébrale doit conduire à réaliser une angiographie cérébrale diagnostique et thérapeutique. La place de la neuroradiologie interventionnelle semble indiscutable en traitement de première ligne. Les indications de sacrifice chirurgical de la carotide se résument actuellement aux échecs du technique endovasculaire.
